# Supplementary material for: VarGenius-HZD Allows Accurate Detection of Rare Homozygous or Hemizygous Deletions in Targeted Sequencing Leveraging Breadth of Coverage
Source: Genes (Basel). 2021 Dec 13;12(12):1979. doi: 10.3390/genes12121979 (PMC8701221; doi:10.3390/genes12121979)
Supplement: Supplementary file 1 [file genes-12-01979-s001.zip › genes-1482188-supplementary.pdf]

Supplementary Material for *VarGenius-HZD allows accurate detection of rare homozygous or hemizygous deletions in targeted sequencing leveraging breadth of coverage*

Supplementary Table S1. HDs called in 1KGP data.
Supplementary Table S2. 1KGP HDs in VarGenius-HZD result.
Supplementary Table S3. 1KGP HDs in ExomeDepth result.
Supplementary Table S4. 1KGP HDs in HMZDelFinder result.
Supplementary Table S5. 1KGP HDs in DECoN result.
Supplementary Table S6. Single nucleotide homozygous variants found in 1KGP samples.
Supplementary Table S7. Summary statistics of HDs found and filtered in the VRCIRD cohort.
Supplementary Figure S1. IGV screenshot of a false positive detected in 1KGP data.
Supplementary Figure S2. Deletion of genes *CFHR1* and *CFHR3* in sample NA20798 of 1KGP data.
Supplementary Figure S3. HD of gene *UGT2B28* in NA18504 1KGP data.

Table S1. HDs called in 1KGP data.

**sample**	**chr**	**position**	**GT**	**Notes**
NA19057	chr12	10581591	1|1:2.000:78,24,0	VGII, ED(filt)
NA19137	chr16	223447	1|1:2.000:133,46,0	VGII (filt), DECoN (filt BF:4.95)
NA18504	chr4	70139616	1|1:1.750:81,13,0	VGII, ED (filt)
NA11919	chr5	140222138	1|1:2.000:573,259,0	VGII,HMZ,ED,DECoN
NA19473	chr9	107366951	1|1:2.000:224,63,0	Putative False Positive

Table S2. 1KGP HDs in VarGenius-HZD result.

**sample**	**BoC**	**DoC**	**AvgDoC**	**chr**	**start**	**end**	**gene**	**1KGP**
NA11919	0,17	8	3214,84	5	140235633	140238168	*PCDHAx*	TP
NA11919	0,18	8	3208,58	5	140235633	140238021	*PCDHAx*	TP
NA11919	0,18	6	2719,3	5	140235633	140237232	*PCDHAx*	TP
NA20798	0	0	178,8	1	196795958	196796135	*CFHRx*	NewTP
NA20798	0	0	162,88	1	196748291	196748486	*CFHRx*	NewTP
NA20798	0	0	152,02	1	196748344	196748486	*CFHRx*	NewTP
NA19057	0	0	52,76	12	10583715	10583827	*KLRCx*	TP
NA18504	0,11	1	87,48	4	70146218	70146939	*UGT2B28*	NewTP
NA19137	0	0	20,72	19	55281302	55281336	*KIRx*	TP(filt)
NA19137	0	0	38,74	16	223470	223599	*HBA2*	TP(filt)

Table S3. 1KGP HDs in ExomeDepth result.

sample	nexons	CNV	BF	reads.expected	reads.observed	reads.ratio	1KGP
NA20798	12	1:196744018-196801129	73,5	337	0	0	NewTP
NA11919	1	5:147553798-147553933	16,4	76	0	0	TP
NA19057	6	12:10583717-10588585	12,6	75	13	0,17	TP
NA18504	6	4:70146220-70160527	22	103	2	0,02	NewTP

Table S4. 1KGP HDs in HMZDelFinder result.

sample	gene	CNV	1KGP
NA11919	*PCDHA10*	5:140235634_140236833	TP
NA20798	*CFHR1*	1:196795959_196796135	NewTP
NA07347	*GHR*	5:42629139_42629205	FP
NA12342	*GHR*	5:42629139_42629205	FP
NA19213	*GHR*	5:42629139_42629205	FP
NA18553	*CES1*	16:55866915_55866967	FP
NA18856	*ZNF630*	X:47918257-47919256	FP
NA19137	*OR5P2*	11:7817521_7818489	FP
NA19236	*OR5P2*	11:7817521_7818489	FP

Table S5. 1KGP HDs in DECoN result.

sample	nexons	CNV	BF	Reads.expected	Reads.observed	Reads.ratio	1KGP
NA19137	2	16:223125-223599	4,59	21	0	0	TP(filt)
NA11919	7	5:140235635-140237232	287	1554	0	0	TP
NA11919	1	5:147553798-147553933	16	75	0	0	TP
NA20798	13	1:196744017-196801129	71,2	327	0	0	NewTP
NA19057	6	12:10583717-10588585	15,8	90	13	0,14	TP
NA18504	7	4:70146220-70160527	23,7	109	1	0,01	NewTP
NA19473	3	9:107379530-107380128	21,9	143	4	0,03	FP

Table S6. Single nucleotide homozygous variants found in 1KGP samples.

<table>
<tr><th>sample</th><th>chr</th><th>position</th><th>ID</th><th>REF</th><th>ALT</th><th>GT</th><th>Notes</th></tr>
<tr><td>NA20798</td><td>chr1</td><td>196733401</td><td>esv2672010</td><td>G</td><td><DEL></td><td>1|1:2.000:239,112,0</td><td>Close HD detected: VG,HMZ,ED,DC</td></tr>
<tr><td>NA20798</td><td>chr1</td><td>196772601</td><td>esv2672625</td><td>G</td><td><DEL></td><td>1|1:2.000:169,82,0</td><td>Close HD detected: VG, HMZ,ED,DC</td></tr>
<tr><td>NA19137</td><td>chr19</td><td>55225201</td><td>esv2671585</td><td>T</td><td><DEL></td><td>1|1:1.550:77,12,0</td><td></td></tr>
<tr><td>NA19137</td><td>chr19</td><td>55280101</td><td>esv2666421</td><td>C</td><td><DEL></td><td>1|1:2.000:92,41,0</td><td></td></tr>
<tr><td>NA19137</td><td>chr19</td><td>55281501</td><td>esv2661467</td><td>C</td><td><DEL></td><td>1|1:2.000:161,63,0</td><td></td></tr>
<tr><td>NA18504</td><td>chr4</td><td>70124301</td><td>esv2656947</td><td>T</td><td><DEL></td><td>1|1:2.000:1495,707,0</td><td>Close HD detected: VG, ED</td></tr>
<tr><td>NA18950</td><td>chr6</td><td>29872907</td><td>esv2659548</td><td>A</td><td><DEL></td><td>1|1:2.000:282,60,0</td><td></td></tr>
</table>

Table S7. Summary statistics of HDs found and filtered in the VRCIRD cohort.

		ED			XHMM			VarGenius-HZD			HMZDelFinder	
	CREv1	CCP	ID	CREv1	CCP	ID	CREv1	CCP	ID	CREv1	CCP	ID
totResults	1744	4542	13797	788	255	168	3100	8916	1162	70	923	1951
totFiltered	52	204	1118	22	18	24	74	745	84	12	76	146
VisButNotFit	0	0	0	1	0	0	3	5	1	3	3	1
FitButNotVis	6	2	0	4	0	0	0	0	1	0	0	0
selPutativeHDs	1	0	0	3	0	0	3	0	3	3	0	2
physicianSelection	1	0	0	0	0	0	3	0	2	3	0	1
Validated-TP	1	0	0	0	0	0	3	0	2	3	0	1
Validated-FP	0	0	0	0	0	0	0	0	0	0	0	0

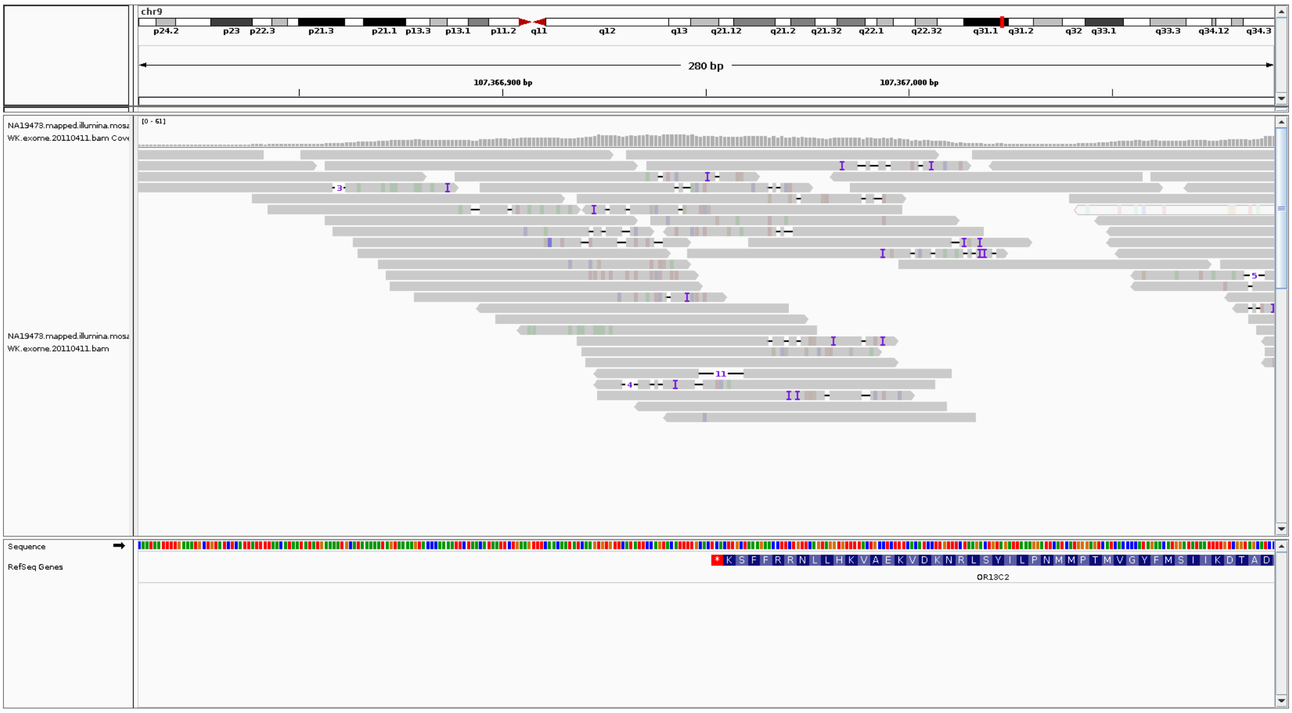

Figure S1. IGV screenshot of a false positive detected in 1KGP data in sample NA19473.

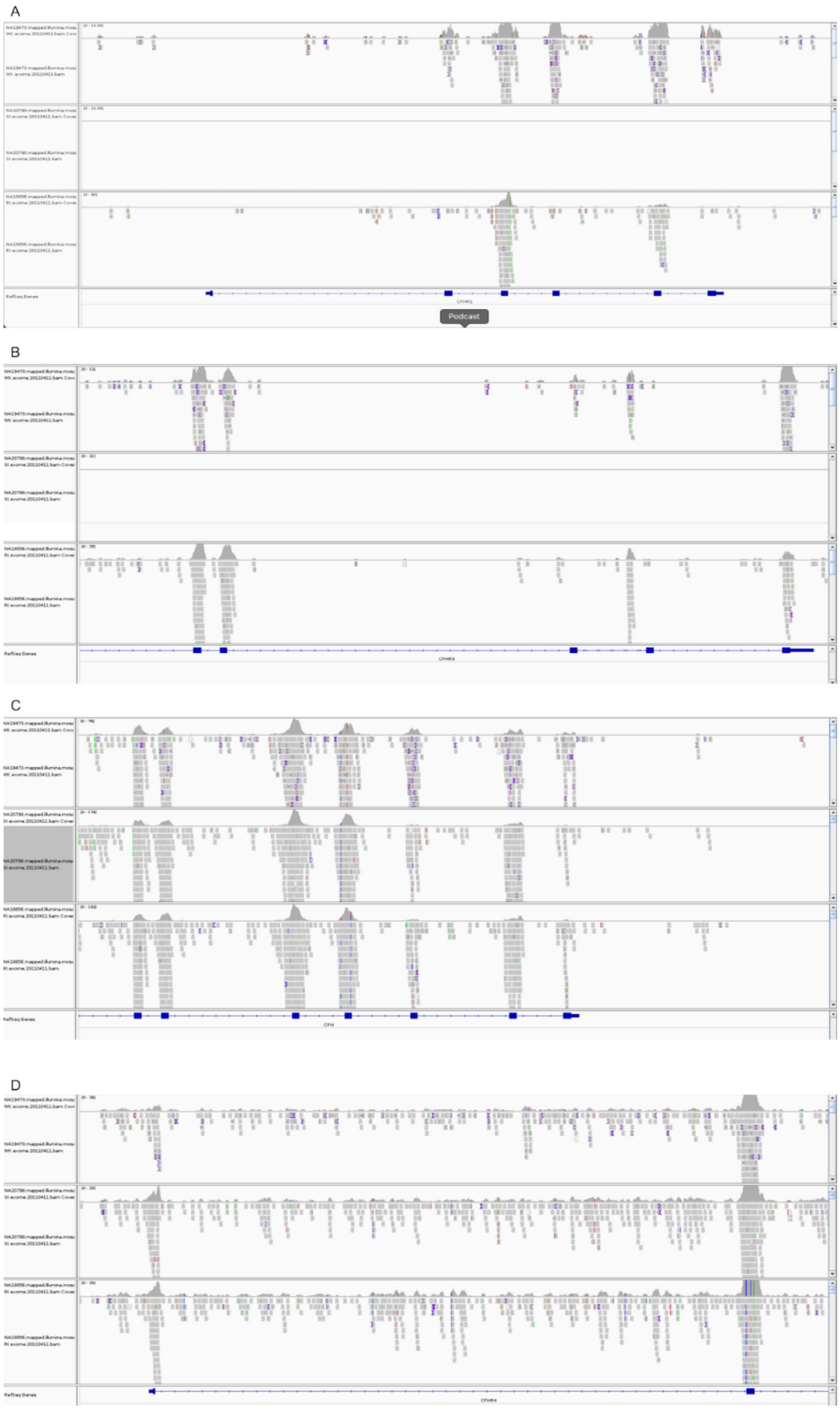

Figure S2. Deletion of genes *CFHR1* and *CFHR3* in sample NA20798 of 1KGP data.

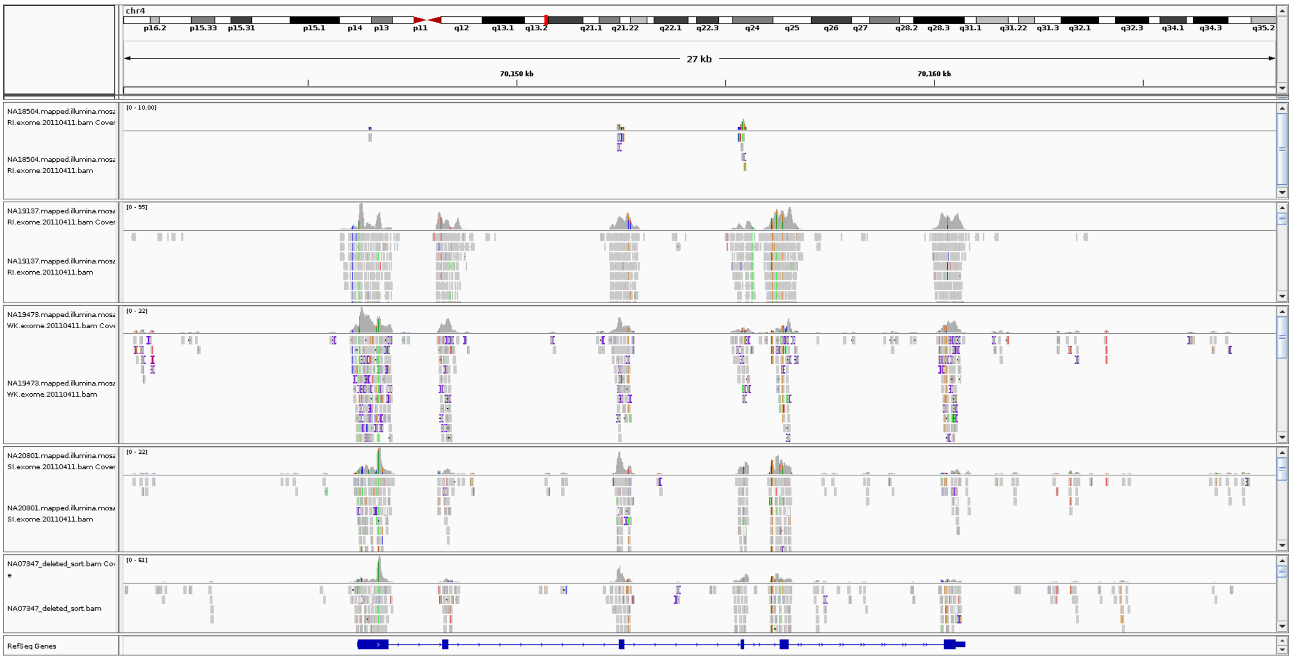

Figure 3. HD of gene *UGT2B28* in NA18504 1KGP data.
